# The association of problematic usage of the internet with burnout, depression, insomnia, and quality of life among Hungarian high school students

**DOI:** 10.3389/fpubh.2023.1167308

**Published:** 2023-07-25

**Authors:** Andrea Feher, Eva Fejes, Krisztian Kapus, Csaba Jancsak, Gabor Daniel Nagy, Lilla Horvath, Antal Tibold, Gergely Feher

**Affiliations:** ^1^Centre for Occupational Medicine, Medical School, University of Pécs, Pécs, Hungary; ^2^Szent Rafael Hospital, Zalaegerszeg, Hungary; ^3^Hospital of Komló, Komló, Hungary; ^4^Centre of Excellence for Interdisciplinary R&D and Innovation of the University of Szeged, Social Responsibility Competence Centre, Interdisciplinary Family R&D Centre Research Group, Szeged, Hungary; ^5^Department of Primary Health Care, Medical School, University of Pécs, Pécs, Hungary

**Keywords:** internet addiction, burnout, depression, insomnia, quality of life, adolescent, student, cross-sectional study

## Abstract

**Introduction:**

The extensive availability of the internet has led to the recognition of problematic usage of the internet (PUI) or so called internet addiction (IA), probably mostly involving adolescents.

**Aim:**

Here we present a study focusing on the incidence and consequences (including burnout, which is relatively rarely studied) of internet addiction among high school students using a questionnaire-based non-random sampling cross-sectional survey. Included questionnaires were the Problematic Internet Use Questionnaire, the Maslach Burnout Inventory General Survey for Students MBI-GS (S), the 9-item short version of Beck Depression Inventory (BDI-SF), the Athens Insomnia Questionnaire and the EQ-5D (quality of life) questionnaire. Data were evaluated the exertion of Student’s *t*-test, chi square test and Pearson’s rank-order correlation. Logistic regression analysis was used to determine the significance of the different parameters as independently associated with PUI.

**Results:**

Overall 3,000 paper-based questionnaires were successfully delivered and 2,540 responses received (response rate of 84.6%). 1,309 males (mean age 17.6 ± 1.43 years) (51.5%) and 1,231 females (mean age 17.5 ± 1.4 years) (48.5%) took part in our study. Problematic usage of the internet was detected in 486 (19.1%) students (232 males, mean age 17.6 ± 1.35 years and 254 females, mean age 17.34 ± 1.37 years). In a logistic regression analysis sleep disturbance (OR: 1.84, 95% CI: 1.83–2.03), depression (OR: 1.97, 95% CI: 1.77–2.02) and burnout (OR: 1.8, 95% CI: 1.16–1.94) were significantly associated with PUI.

**Conclusion:**

Nearly one fifth of our study population suffered from PUI, which was strongly associated with school burnout, insomnia and depression, which underlines the importance of this phenomenon.

## Introduction

The fact that the internet become easily accessible and affordable has radically changed our lives by the end of the 20th century and has become an integral part of everyday life including both recreation and work. Apart from favourable effects such as rapid information flow and easy access to global databases, its use also has downsides, including problematic usage of internet (PUI), which is considered a new type of addiction (1–3).

The biggest obstacle to recognizing this phenomenon is the extent to which the internet has penetrated our everyday lives; in the vast majority of cases neither the person nor their environment recognizes misuse and/or its warning signs (2–5). The phenomenon is in the focus of multidisciplinary scientific research generating quarrels and debates among scientists and the public (2–5). It has been referred to many nomenclatures including internet addiction, pathological, maladaptive, excessive, problematic or compulsive internet use, problematic usage of the internet, the current nomenclature prefers the usage of PUI (2, 5–7).

The assessment of problematic internet use is uniform despite different points of view: problematic internet use is defined as problematic use or abuse (apart from its goal), which – similar to „classic” addictions - results in severe impairments with regard to the individual’s daily activities in various life domains over a prolonged period of time (2, 3). Based on current knowledge there are two main subtypes of PUI, the first type is related to impulsive behaviours such as gaming or gambling, the second type seems to be related to compulsive behaviours such as cyberchondria or digital hoarding, but is should be mentioned that significant overlap exists (7).

The situation is complicated by lack of proper diagnostic criteria of problematic usage of the internet, only gaming disoder was listed as a candidate disoder warranting further investigation in the latest DSM-5 classification (Diagnostic and statistical manual of mental disorders, 5th edition) (7, 8). Gaming disorder (together with gambling disoder) was defined as a full diagnosis in the latest ICD-11 (International statistical classification of diseases and related health problems), both predominantely ‘online’ and ‘offline’ forms as well as ‘undefined’ group were mentioned (8, 9). Regardless, it can be mentioned that the definition of problematic usage of the internet and its possible subtypes is far from complete.

Problematic usage of the internet can affect approximately 7% of the whole population based on the results of a recent meta-analysis, however, increasing prevalence rates have been reported year by year, but usually based on non-representative samples, which should be treated with caution as they rather cover frequencies in specific populations than overall prevalence (2, 7, 10). In Hungary based on data from the Central Statistical Office and Gemius and also provided by Ipsos surveys the total number of Internet users doubled and the time spent online had and eight-fold increase within 15 years, which presumably increased the rate of problematic usage (11–13). A relatively recent representative study showed that the prevalence of PUI varies between 1%–10% in the general population, but the possible modifying factors were not mentioned (14). The prevalence can vary between 10%–30% and probably negatively correlates with age among the youth and our recent studies showed that its frequency can be approximately 5% in specific adult populations, but these data are far from conclusive (3, 15–17).

Pioneer studies showed that young people (<25 years) are most often affected. Its prevalence can be as high as 20% among them; the younger the use of digital devices, the greater the likelihood to develop an addiction, consequently, this vulnerable group is usually the focus of investigations (2, 5, 6). The older adult population (especially those ≥50 years) have been rarely studied from this point of view, but these results showed that in addition to age the purpose of internet use is also an important factor in the development of PUI (7, 18). The use of ‘time wasters’ or watching streams can enhance PUI among middle-aged or older ones, while watching pornography is more frequent among younger individuals (18).

Male gender has generally been considered to increase the risk of problematic use, probably due to males being open towards technological innovations, but there are significant discrepancies in the existing literature (2, 7, 18, 19). Male gender may predisponate to problematic online gaming or online pornography, while abusive social media use or smarphone addiction are more frequently associated with female gender (7, 20).

Relationships with family members and its quality also plays a role in the development of compulsive internet use. More superficial, less emotional child–parent relationships and common family conflicts can increase the rate of turning to the online space (21). Low family income, and living in rural areas also seem to be important demographic factors (22). The lack of social support in school or at workplace can also be important. Being a victim of cyberbullying can be an important predecessor of PUI as well as having less personal contacts at school (23).

Apart from demographic and socioeconomic factors, several personality traits such as higher impulsivity, lower self-control and sensation seeking as well as being socially inactive and neuroticism can unbalance the ratio of daily activities in favour of being online (24). A recent meta analysis including 37 studies with more than 34,000 participants (vast majority from Asia) showed that neuroticism and psychoticism are probably the most important risk factors, while being untruthful, agreeable, conscientious and extraverted can be protective against PUI (25).

Internet addiction can be associated with a cluster of mental problems including anxiety, agressive behaviour, social anxiety, suicidal intention, depression, attention deficit hyperactivity disorder (ADHD) and autism, the relationship seems to be the closest in the case of depression and ADHD (7, 26–28). The association of internalizing disorders (i.e., depression) and PUI can be bidirectional (7). The first hypothesis is that individuals with negative emotions might find online recreational activities to relieve stress or avoid anxiety emotions (so-called negative-reinforcing motivations) (7, 29). Whatever is the reason turning for internet (either a specific personality trait or mood imbalace) it does not automatically or fully compensate for offline failure(s) (23). On the other hand, online (fake) comparisms such as image based social media can generate negative thoughts and feelings resulting in anxietey and depression (7, 23, 29). Moreover, comparisms to perfectly polished pictures or fittness contents can result in other mental issues including body image disturbances and eating disorders (7).

Based on currents studies the rate of mood disorder/depression is increased among problematic users (there is an approximately 2.5-fold risk) and considering that anxiety is often intertwined with depression, it is not surprising that it is also associated with online addictions, but the association should be clarified as seen above (30–32). One of the key points of the association of PUI and depression can be sleeping problems (33, 34). The duration of sleep (reducing sleeping time for online activities and tendency to sleep late) and its quality (sleep problems including insomnia) seems to be impaired among those with IA due to irregular sleep patterns with consequent fatigue daytime sleepiness. A recent meta-analysis revealed a two-fold risk in the development of insomnia (33). Similar to internalizing disoders, impulsive problems are strongly associated with PUI (7, 34, 35). Impulsivity, being easily bored and aspiration for achieving immediate success both are key components of ADHD and PUI (7, 24, 35). However, in the case of ADHD the causality is still unkown similar to the connection between PUI and depression.

Based on the above mentioned findings it is not suprising that there is a negative correlation between PUI and quality of life as summerized by a recent meta analysis (36). In general, PUI seems to be associated with lower health-related quality of life and seems most likely related to severe mental issues as seen above (36, 37). It is worth to mention that nations with greater pollution and traffic time comsumption as well as with lower satisfaction with life have a greater ratio for PUI, which also underlines the role of negative quality of life (21).

Only few studies have focused on the association school or workplace burnout and IA among adolescents (and adults). Similar to IA, burnout is also a rapidly increasing phenomenon with diverse etiologies including both organisational and individual traits (38). We also have to face with a lack of evidence-based classification of burnout (as well as in the case of IA). Based on the generally accepted theory it can be characterised as a triad of symptoms including emotional exhaustion, depersonalization and (reduced) personal accomplishment (2, 3, 31). Initially, work-related stress was considered to be the main determinant of burnout, but recent studies have also emphasized the role of personality traits such as pliancy and neuroticism (2, 3, 32, 39). Even though the concept of burnout was developed primarily in a workplace context, this phenomenon can also apply to educational (and parental) settings as similar to work, being educated (or having children) requires individuals to engage with multiple achievement pressures (40). In this particular case school burnout may be defined as a response to school-related stress, which may also result from internal and external factors alike (2, 3, 41, 42).

Although burnout is still not considered as a medical condition apart from its great impact on the individual’s work (lack of energy, reduced coping skills, emotional depletion etc.), it seems to be strongly be associated with severe mental and somatic complications including mood disorders, unfavourable cardiovascular outcomes and chronic pain syndromes (2, 3, 40). There is a strong link between burnout and substance abuse (tobacco use, alcohol consumption or taking drugs) and recent studies have raised the possibility of subsequent problematic internet use as a behavioural addiction (2, 3, 43). IA can lead to consequent school burnout and vice versa based on a cross-sectional study among Finnish adolescents (42).

As seen above PUI is a complex phenomenon and many factors can play a role in its development. However, studies so far have mainly focused on several factors factors and used different methodological approaches. Our present study is part of a larger research focusing on the prevalence of internet addiction and its risk factors (taking many covariates into account) as well as its relationship with mental issues (namely insomnia, depression, burnout) and overall quality of life using a similar methodology in different Hungarian populations to make comperable results. Some studies of our research have already been published (2, 3, 16, 44). There are relatively few similar complex research available in our country.

We have previously examined the prevalence and risk factors of problematic internet use on the same population (among Hungarian high-school students) and here we present the second part of our cross-sectional study focusing on the association of PUI with mental issues and quality of life also taking the pontential role of the previously published parameters into account.

## Materials and methods

This cross-sectional paper-based questionnaire study was conducted between 04/2019 and 03/2020 in 8 large educational sites in Middle- and South Hungary (see Acknowledgements) with a non-probability sampling method. Questionnaires were divided amongst study sites, were handled by the students’ teachers and completed forms were later gathered. The study protocol was approved by the Ethical Committee of the University of Pecs (8434-PTE 2020) and was also approved and funded by the National Research, Development and Innovation Office as part of a larger research project as mentioned before (NKFI-135316 project) (45). Signed consent was obtained from the respective school authorities prior to data collection. Informed consent was signed by participants (and by parents or guardians if students were <18 years) before fulfilling the questionnaire.

The inclusion criteria were being enrolled as a student during the period of this study, being willing to participate and having signed, informed consent by the participant or by parents or guardians if the participant was younger than 18 years of age.

The included *demographics, risk factors, previous diseases* and *details of internet* use had been previously published and can be seen in Supplementary Table 1 (2).

*Problematic usage of the internet* was detected with the Problematic Internet Use Questionnaire, which was originally developed by Demetrovics and his co-workes in Hungarian language and have been translated (and validated) to other languages ever since (Cronbach’s alpha 0.912) (2, 3, 46–48). The questionnaire contains 18 items, which can be divided into three main parts namely obsession, neglect and control disoder. Obsession subscale refers to obsessive thinking about the Internet (daydreaming, rumination, and fantasizing) and withdrawal symptoms caused by the lack of Internet use (anxiety and depression) (“How often do you feel tense, irritated, or stressed if you cannot use the Internet for as long as you want to?”). Neglect subscale contains items about neglecting everyday activities, social life, and essential needs (“How often do you spend time online when you’d rather sleep?”). Control disorder subscale reflects difficulties in controlling time spent on the Internet (“How often do you realize saying when you are online, “just a couple of more minutes and I will stop”?”). Each of them consists of six questions. The answers are scored on a 5-point Likert-type scale ranging from 1 (never) to 5 (always). A total score exceeding 41 points suggests Internet addiction (2, 3, 49).

*Burnout* was measured with the Maslach Burnout Inventory General Survey for Students MBI-GS (S), which is a reliable instrument of school burnout (2, 3, 50, 51) (Cronbach’s alpha 0.819). This validated 15-item instrument has three subscales according to the most widely accepted theory of burnout and these are emotional exhaustion (being overburdened and exhausted by one’s studies), cynicism (distant attitude towards studies) and professional efficacy (satisfaction with past and present accomplishments). The items refer to the burdensome feeling of education within the last 3 months, for example: “I feel exhausted by the end of a day spent at school.” Responses are marked on a 7-point Likert scale from 0 (meaning ‘never’) to 6 meaning (‘every day’) and then summed. High scores on cynism and emotional exhaustion while low scores on professional efficacy are indicative of burnout (2, 3, 50, 51).

The presence of *depression* was detected by the 9-item short version of Beck Depression Inventory (BDI-SF) (Cronbach’s alpha 0.819). The questionnaire contains questions on the following symptoms: indecision, social withdrawal, fatigue, sleep disturbance, incapacity for work excessive anxiety about physical symptoms, pessimism, lack of joy, dissatisfaction and self-blame. Each answer is scored on a 4-point Likert scale from 1 to 4 points (2, 3, 52, 53). Depression can be detected over 9 points and we can distinguish between mild (10–18 points), moderate (19–25 points) and severe depression (≥26 points) (36). This questionnaire is available and validated in Hungarian language (2, 3, 53).

*Sleep disturbace* was assessed with the Athens Insomnia Questionnaire, which contains 8-items focusing on nocturnal symptoms (5 items) and daytime sleepiness (3 items) (Cronbach’s alpha 0.813) (37). Each answer is rated on a 4-point Likert scale from 0 to 3. Reaching >6 points means the presence of insomnia, while >10 points means clinically significant, severe insomnia (2, 3, 54, 55). This questionnaire is also available in Hungarian language (2, 3, 55).

*Quality of life* was detected with the EQ-5D questionnaire, which is a valid, standardized instrument for measuring generic health status translated into many languages (Cronbach’s alpha 0.742) (56). This is also a self-administered questionnaire focusing on 5 dimensions of everyday life (self-sufficiency, mobility, pain/malaise, normal daily activities and anxiety/depression) with the inclusion of a Likert-scale ranging from 0 (no problem at all) to 5 (very severe problem/disability) (2, 3).

*Statistical analysis*: data were evaluated as means ± Standard Deviation (SD) with the exertion of Student’s *t*-test, chi square test and Pearson’s rank-order correlation (*n* = 2,540). Logistic regression analysis was used to determine the significance of the different parameters as independently associated with PUI. The analysis included those parameters found to be significant in a univariate analysis (see above). The analysis was performed with appropriate adjustments for differences in risk factors and medication usage. For all odds ratios, an exact confidence interval (CI) of 95% was constructed in our study. Data analysis was performed using SPSS (version 22.0, IBM, New York, NY, United States) (2, 3).

## Results

Overall, 3,000 paper-based questionnaires were successfully delivered and 2,540 responses received (response rate: 84.6%). 1,309 males (mean age 17.6 ± 1.43 years) (51.5%) and 1,231 females (mean age 17.5 ± 1.4 years) (48,5%) took part in our study. Baseline characteristics had previously been published and can be seen in Supplementary Table 1 (9).

Problematic usage of the internet was detected in 486 (19.1%) students (232 males, mean age 17.6 ± 1.35 years and 254 females, mean age 17.34 ± 1.37 years) based on the Problematic Internet Use Questionnaire.

With regards demographic data, risk factors and comorbidities, in our previously published study age, living without parents, household containing >5 people, being online ≥6 h daily, daily time interval were associated with PUI as well as alcohol consumption, drug intake, history of musculoskeletal pain and depression (Supplementary Table 2) (2).

In our study population, 520 (20.5%) suffered from mild, 1,690 (66.5%) from moderate, and 330 (13.0%) from severe burnout based on the Maslach Burnout Inventory. Regarding the subcategories, mean values were the following: emotional exhaustion (2.89 ± 1.26 points; 95% CI 2.84–2.94), depersonalization (2.98 ± 1.17 points; 95% CI 2.94–3.03), personal accomplishment 2.59 ± 1.37 points; 95% CI 2.54–2.64 points.

School burnout was also related to PUI, the rate of severe burnout was significantly higher in the PUI group (21.8 vs. 10.9%, *p* < 0. 001). Emotional exhaustion (2.33 ± 0.34 vs. 3.34 ± 0.17, *p* < 0.001) and depersonalization (1.75 ± 0.11 vs. 2.72 ± 0.25, *p* < 0.001) were positively associated with PUI (Table 1). However, the highest quartile of reduced personal accomplishment had the lowest internet addiction scores (not shown).

**Table 1:** Comparison of depression.

*N* = 2,540	Not addicted to the internet (*n* = 2054)	Internet addiction (*n* = 486)	df	*p*
Depression			**1,272**	
No depression	391 (19.1%)	18 (3.7%)		0.164
Mild	1,508 (73.4%)	313 (64.4%)		0.210
Moderate	147 (7.1%)	142 (29.2%)**		0.000
Severe	8 (0.4%)	13 (2.7%)**		0.000
Sleep disturbance (insomnia)			**1,166**	
No	1,393 (67.8%)	176 (36.2%)		0.173
Present	479 (23.3%)	169 (34.8%)**		0.000
Severe	182 (8.9%)	141 (29%)**		0.000
Burnout			**4,399**	
Low	460 (22.4%)	60 (12.4%)		0.126
Moderate	1,370 (66.7%)	320 (65.8%)		0.085
Severe	224 (10.9%)	106 (21.8%)**		0.000
Depersonalization	1.75 ± 0.11	2.72 ± 0.25**		0.000
Emotional exhaustion	2.33 ± 0.34	3.34 ± 0.17**		0.000
Personal accomplishment	3.53 ± 0.45	3.03 ± 0.4		0.952
Quality of life (points)				
	81.4	75.5**	**3,869**	0.000

Sleep disturbance. Burnout and quality of life in the study subgroups (**p < 0.001).

Depression could not be detected in 16.1% (410/2540) of the participants, while 71.7% (1811/2540) had mild 11.4% (299/2540) had moderate and 0.8% (20/2540) had severe depression based on the results of BDI. The rate of moderate (29.2 vs. 7.1%) and severe (2.7% vs. 0.4%) depression were also significantly higher in the PUI group (*p* < 0.001).

Insomnia could be found in 25.5% (648/2540) of the study population, while 12.7% (323/2540) suffered from severe sleep disturbance. Problematic usage of the internet was associated with insomnia, the presence of sleep disturbance (34.8% vs. 23.3%) and severe imsomnia (29% vs. 8.9%) were significantly higher in the IA group (*p* < 0.05) (Table 1).

Problematic usage of the internet was also associated with lower quality of life based on the EQ-5D Questionnaire (75.5 points, 95% CI 73.3–77.6 vs. 81.5 points; 95% CI 80.5–82.2, *p* > 0.001), mostly driven by impaired daily activity (0.79 ± 0.39 vs. 0.46 ± 0.14, *p* = 0.004), pain (0,60 SD = 0.86 ± 0.6 vs. 0.68 ± 0. 35, *p* = 0.004) and dysphoria (0.61 SD = 0.89 ± 0.61 vs. 0.63 ± 0.28, *p* = 0.002) (Table 1).

A logistic regression performed to ascertain the effects of previously found predictors such as age, living without parents, household contatining 5 persons or more, being online ≥6 h, daily time interval, alcohol consumption, drug intake, history of musculoskeletal pain and depression, as well as the currently studied burnout, current depression, insomnia and quality of life on the likelyhood of the presence of burnout versus the absence of this phenomenon. The logistic regression model was statistically significant (*p* = 0.000, *X*^2^ = 31.85). The model explained 5.8% (Nagelkerke *R*^2^) of variance in burnout and correctly classified 72.0% of the cases.

Logistic regression analysis showed that apart from age (OR 1.49; 95% CI: 1.22–2.08), smoking (OR: 1.47, 95% CI: 1.44–2.25), drug comsumption (OR: 1.91, 95% CI: 1.15–1.99), history of musculoskeletal disorders (OR: 1.38, 95% CI: 1.14–3.71), being online ≥6 h (OR: 1.73, 95% CI: 1.717–2.04), daily time interval (OR: 1.72, 95% CI: 1.56–1.96) PUI was also significantly associated with sleep disturbance (OR: 1.84, 95% CI: 1.83–2.03), depression (OR: 1.97, 95% CI: 1.77–2.02), and burnout (OR: 1.8, 95% CI: 1.16–1.94) (Table 2).

**Table 2:** Factors independently associated with IA in a logistic regression analysis.

*N* = 2,540	B	SE B	*p*	OR	CI 95% lower	CI 95% upper
Age*	0.01	0.37	0.027	1.49	1.22	2.08
Family status	0.05	0.15	0.099	0.21	0.78	1.42
Number of persons sharing a household	0.02	0.15	0.086	0.22	0.76	1.37
Smoking*	0.03	0.39	0.014	1.47	1.44	2.25
Drug consumption*	0.95	0.48	0.003	1.91	1.15	1.99
Musculoskeletal disorders*	1.12	1.57	0.041	1.38	1.14	3.71
Time spent ≥ 6 h online*	0.16	0.09	0.000	1.73	1.717	2.04
Daily time interval*	1.32	1.94	0.006	1.72	1.56	1.96
Sleep disturbance*	0.08	0.05	0.000	1.84	1.83	2.03
Burnout*	0.14	0.042	0.009	1.8	1.16	1.94
Depression*	0.01	0.013	0.001	1.97	1.77	2.02

## Discussion

This is among the first and most comprehensive studies from Hungary showing the assoctiation of problematic usage of the internet with school burnout and mental issues. The rate of problematic internet users were more common than the estimated 7%, but recent studies have also revealed a constantly increasing rate (2, 21, 57).

PUI was associated with school burnout in our population, which is rarely studied in the context of problematic internet use. Although both burnout and problematic internet use are considered to be serious mental health problems among adolescents, they are still labelled as phenomena and not as medical conditions (58). Both are strongly related to chronic stress, and are associated with negative effects on social relationships, educational performance, physical, and psychological wellbeing (2, 3, 58, 59).

The association can be bidirectional and a cross-sectional study is unsuitable to reveal the exact relationship. First of all, burnout can lead to addictive behaviour such as substance abuse or internet gaming disorder. The other explanation is that lack of social skills, school problems etc. can lead to the excessive and problematic use of internet aggravating the existing social and learning problems resulting in school burnout (2, 58).

Depersonalization, which is one of the main components of burnout in the currently establihed three-way model and found to be associated with PUI in our study can result in weaker communication or social skills and addictions (such as PUI) can be the consequences of trying to escape from real problems and coping with psychosocial stress (60). Emotional exhaustion, which is generally considered as the most important predecessor of burnout was also associated with problematic usage of the internet based on our results, which could be related to the fact that being emotionally unstable can result in higher anxiety levels, and reduced communications skills with subsequent social isolation (58, 60, 61). These people also tend to be imprisoned in the world of the internet further deteriorating the underlying behavioral abnormality (2, 3). Interestingly, reduced personal accomplishment seemed to be a protective factor in the development of PUI. It has been shown that decline in personal accomplishment is usually less related to a stress factor and is probably not related to inadequate coping mechanisms facilitating an ability to resist problematic internet use. We have no clear explanation to this phenomena, however, similar results have been found in a Japanese study (60).

Nevertheless, problematic usage of the internet was independently associated with burnout in a multivariate analysis drawing our attention to the complexity of its background. Moreover, altered function of higher-order brain networks may explain this association as both volumetric changes and alteration of task-related activities can be detected in similar brain areas, but the association merits further investigation (2, 3, 62–65).

PUI was also associated with mental issues such as sleep disturbance and depression in both uni- and multivariate analysis. The association of PUI and psychiatric symptoms is not well understood (61). An underlying psychopathology (anxiety/mood disorder or poor sleep quality) can facilitate turning to the internet to ease problems or problematic internet use may lead to the development of impaired sleep and mood disorders, and finally, it is also possible that they enhance each other (2, 3, 66). Insomnia may be due to the consequence of PUI as has been indicated by results of previous studies and has also been supported by our previously published results on the same population showing the role of nighttime internet use as a significant risk factor for addiction, but these results are more speculative than scientific in this particular case (2, 3, 67).

PUI was also associated with increased rates of moderate and severe depression, which underlines the importance of proper screening as depression is projected to be the leading cause of disability by 2030 and severe mood disorders are disabling states for both the individual and society (2, 68). Although the causality is not entirely known, similar to depression, PUI has also been associated with markedly increased rates suicidal ideation and planning, which suggests that we should labelled PUI as a medical condition and it should not be considered as a temporary phenomena due to the well-known mental instability of adolescents (69). Finally, it should be mentioned that there seems to be a significant overlap in the symptomatology of both burnout and depression, but a recent moderation analysis identified them as different conditions and our results also confirmed their separate roles as significant factors associated with PUI in a multivariate analysis (70).

Due to the widespread negative effects of PUI on various life domains, it is not suprising that it is associated with lower quality of life. However, the association is relatively poorly studied compared to the increasing rates of research carried out on the topic. A recent meta analysis including only 18 papers focusing on the above association found that, similar to our results, problematic internet use was associated with lower overall quality of life as well as lower psychological and physical health (36). In our study population, PUI was previously found to be associated with living without parents, nighttime internet use, alcohol and drug comsumption and history of musculoskeletal pain and currently we found a strong relationship among problematic internet use with depression, insomnia and burnout (2). The previously mentioned parameters could be responsible for the reduced quality of life; however; quality of life was not significantly associated with IA in a multivariate analysis, which merits further investigation. It is worth to mention that nations with greater pollution and traffic time comsumption as well as with lower satisfaction with life have a greater ratio for PUI and it is more important in the development of the phenomena than the extent of internet availability, which also underlines the role of negative quality of life (21).

Finally, our article has some limitations. Due to the lack of standardized methodology the definition of problematic usage of the internet is still a matter of debate. This study was soon before carried out the first outbrake of COVID-19 in Hungary, which may affect our results. Although it was a prospective study in nature including more than 2,500 students, it was not representative of internet addiction neither in the general nor in the adolescent population and the applied non-probability sampling increase the risk of survey bias. The definition of PUI, insomnia and mental issues were based on the results of the survey questionnaires, which does not automatically mean a proper clinical diagnosis. In our study neither physical nor psychological examination was not carried out and we also had no detailed information about the medical history of our participants, medical data were extracted from the applied surveys. Finally, follow-up was not carried out. The above mentioned limitations may have influenced our findings.

In general, our study is one of the first and most comprehensive reports from Hungary showing the association of PUI with mental issues and burnout. Approximately 1 out of five student suffered form PUI, which is above the expected 7%. It may be advisable to develop appropriate school prevention procedures, such as limited access or blocked internet during school hours as well as implementing appropriate prevention education. The results of our current study raise the possibility of looking for problematic internet use in the background of reduced school performance and associated sleep disorders, depression and school burnout, which are closely related. Our results may change the current viewpoint of the phenomenon and may highlight its potentially serious consequences.

## Data availability statement

The raw data supporting the conclusions of this article will be made available by the authors, without undue reservation.

## Ethics statement

The studies involving human participants were reviewed and approved by The study protocol was approved by the Ethical Committee of the University of Pecs (8434-PTE 2020). Written informed consent to participate in this study was provided by the participants’ legal guardian/next of kin.

## Author contributions

AF, EF, KK, CJ, GN, LH, AT, and GF equally contributed to the manuscript including study concept and design, collection of data, analysis and interpretation of data, writing of manuscript, and critical revision of manuscript. All authors contributed to the article and approved the submitted version.

## Funding

This research was funded by NKFI (OTKA)-135316 project.

## Conflict of interest

The authors declare that the research was conducted in the absence of any commercial or financial relationships that could be construed as a potential conflict of interest.

## Publisher’s note

All claims expressed in this article are solely those of the authors and do not necessarily represent those of their affiliated organizations, or those of the publisher, the editors and the reviewers. Any product that may be evaluated in this article, or claim that may be made by its manufacturer, is not guaranteed or endorsed by the publisher.
