# Supplementary material for: The association of problematic usage of the internet with burnout, depression, insomnia, and quality of life among Hungarian high school students
Source: Front Public Health. 2023 Jul 25;11:1167308. doi: 10.3389/fpubh.2023.1167308 (PMC10407570; doi:10.3389/fpubh.2023.1167308)
Supplement: Supplementary file 1 [file Table_1.docx]

| ***Study population (number)*** | **2540** |
| --- | --- |
| *Mean age (years)* | 17.56 ± 1.41 |
| *Males (number)* | 1309 (51.5%) |
| *Mean age (years)* | 17.6 ± 1.43 |
| *Females (number)* | 1231 (48.5%) |
| *Mean age (years)* | 17.5 ± 1.4 |
| *Age distribution* | |
| 15 | 99 (3.9%) |
| 16 | 498 (19.6%) |
| 17 | 678 (26.7%) |
| 18 | 584 (23.0%) |
| 19 | 437 (17.2%) |
| 20 | 177 (6.96%) |
| 21 | 46 (1.8%) |
| 22 | 21 (0.84%) |
| *Family type* | |
| married parents | 1465 (57.7%) |
| parental civil partnership | 279 (11.0%) |
| single parent | 627 (24.5%) |
| fosterer | 86 (3.4%) |
| students living with partner | 46 (1.8%) |
| children’s home | 25 (1.0%) |
| other (none of the above mentioned types) | 12 (0.5%) |
| *Type of residence* | |
| house | 1857 (73.2%) |
| flat | 472 (18.6%) |
| farm | 177 (6.9%) |
| institution | 28 (1.1%) |
| other (none of the above mentioned types) | 6 (0.2%) |
| *Number of persons sharing a household* |  |
| 1 | 23 (0.9%) |
| 2 | 226 (8.9%) |
| 3 | 631 (24.8%) |
| 4 | 865 (34.1%) |
| 5 | 503 (19.8%) |
| > 5 | 292 (11.5 %) |
| *Concomitant diseases* | |
| taking medication regularly | 222 (8.8%) |
| smoker | 578 (22.7%) |
| taking alcohol more or less regularly | 262 (10.3%) |
| taking drugs more or less regularly | 237 (9.3%) |
| diabetes | 48 (1.9%) |
| hypertension | 199 (7.9%) |
| cardiovascular disease | 94 (3.7%) |
| musculoskeletal pain | 40 (1.6%) |
| depression or other psychiatric disease | 48 (1.9) |

Supplementary Table 1. Baseline characteristics of the study population

| **N=2540** | Not addicted to the internet (n= 2054) | Internet addiction (n= 486) | p | df |
| --- | --- | --- | --- | --- |
| *Gender* | | |  | *106* |
| *Males* | *1077 (52.4%)* | *232 (47.7%)* | *0.137* |  |
| *Females* | *977 (47.6%)* | *254 (52.3%)* | *0.126* |  |
| *Age (years)* | | |  | *371* |
| 15 years | 79 (3.8%) | 20 (4.1%) | 0.176 |  |
| 16 years | 414 (20.1%) | 84 (17.3%) | 0.060 |  |
| 17 years | 500 (24.3%) | 178 (36.6%)** | 0.000 |  |
| 18 years | 485 (23.6%) | 99 (20.4 %) | 0.160 |  |
| 19 years | 366 (17.8%) | 71 (14.6 %) | 0.321 |  |
| 20 years | 152 (7.4%) | 25 (5.1 %) | 0.096 |  |
| 21 years | 38 (1.8%) | 8 (1.7%) | 0.185 |  |
| 22 years | 20 (0.9%) | 1 (0.2%) | 0.884 |  |
| *Family type (%)* | | |  | *424* |
| married parents | 1175 (57.2%) | 290 (59.7%) | 0.477 |  |
| parental civil partnership | 231 (11.3%) | 48 (9.9%) | 0.288 |  |
| single parent | 518 (25.2%) | 104 (21.4%) | 0.227 |  |
| fosterer | 66 (3.2%) | 20 (4.1%)* | 0. |  |
| students living with partner | 40 (1.9%) | 5 (1%) | 0.330 |  |
| children’s home | 15 (0.7%) | 10 (2%)* | 0.017 |  |
| other (none of the above mentioned types) | 9 (0.4%) | 9 (1.8%)* | 0.008 |  |
| *Type of residence (%)* | | |  | *265* |
| house | 1505 (73.2%) | 352 (72.3%) | 0.926 |  |
| flat | 378 (18.4%) | 94 (19.4%) | 0.830 |  |
| farm | 148 (7.2%) | 29 (6%) | 0.916 |  |
| institution | 18 (0.9%) | 10 (2%) | 0.699 |  |
| other (none of the above mentioned types) | 5 (0.3%) | 1 (0.2%) | 0.593 |  |
| *Number of persons sharing a household (%)* | | |  | *265* |
| 1 | 15 (0.7%) | 6 (1.2%) | 0.093 |  |
| 2 | 192 (9.3%) | 37 (7.6%) | 0.806 |  |
| 3 | 522 (25.4%) | 109 (22.4%) | 0.829 |  |
| 4 | 698 (34%) | 167 (34.4%) | 0.473 |  |
| 5 | 410 (20%) | 93 (19.1%) | 0.366 |  |
| > 5 | 218 (10.6%) | 74 (15.2%)* | 0.024 |  |
| *Concomitant diseases* | |  |  | 53 |
| taking medication regularly | 180 (8.7%) | 42 (8.6%) | 0.262 |  |
| smoker | 443 (21.6%) | 135 (27.7%)* | 0.008 |  |
| taking alcohol | 195 (9.4%) | 67 (13.7%)* | 0.023 |  |
| taking drugs | 176 (8.5%) | 61 (12.5%) | 0.716 |  |
| diabetes | 40 (1.9%) | 9 (1.8%) | 0.290 |  |
| hypertension | 159 (7.7%) | 40 (8.2%) | 0.059 |  |
| cardiovascular disease | 70 (3.4%) | 25 (5.1%) | 0.765 |  |
| musculoskeletal pain | 31 (1.5%) | 11 (2.2%)* | 0.009 |  |
| depression or other psychiatric disease | 39 (1.8%) | 12 (2.4%)* | 0.008 |  |
| *Daily internet use (approximately)* | | |  | *424* |
| 1 hour | 127 (6.2%) | 9 (1.8%) | 0.878 |  |
| 2 hours | 371 (18%) | 47 (9.7%) | 0.947 |  |
| 3 hours | 472 (23%) | 79 (16.3%) | 0.768 |  |
| 4 hours | 420 (20.4%) | 79 (16.3%) | 0.686 |  |
| 5 hours | 257 (12.4%) | 71 (14.6%) | 0.394 |  |
| 6 hours | 114 (5.6%) | 45 (9.2%)** | 0.000 |  |
| > 6 hours | 293 (14.3%) | 156 (32.1%)** | 0.000 |  |

Supplementary Table 2. Comparison of internet use in the study subgroups.

| **N=2540** | Not addicted to the internet (n= 2054) | Internet addiction (n= 486) | **df** | **p** |
| --- | --- | --- | --- | --- |
| **Depression** | |  | **1272** |  |
| no depression | 391 (19.1%) | 18 (3.7%) |  | 0.164 |
| mild | 1508 (73.4%) | 313 (64.4%) |  | 0.210 |
| moderate | 147 (7.1%) | 142 (29.2%)** |  | 0.000 |
| severe | 8 (0.4%) | 13 (2.7%)** |  | 0.000 |
| **Sleep disturbance (insomnia)** | | | **1166** |  |
| no | 1393 (67.8%) | 176 (36.2%) |  | 0.173 |
| present | 479 (23.3%) | 169 (34.8%)** |  | 0.000 |
| severe | 182 (8.9%) | 141 (29%)** |  | 0.000 |
| **Burnout** | | | **4399** |  |
| low | 460 (22.4%) | 60 (12.4%) |  | 0.126 |
| moderate | 1370 (66.7%) | 320 (65.8%) |  | 0.085 |
| severe | 224 (10.9%) | 106 (21.8%)** |  | 0.000 |
| depersonalization | 1.75 ± 0.11 | 2.72 ± 0.25** |  | 0.000 |
| emotional exhaustion | 2.33 ± 0.34 | 3.34 ± 0.17** |  | 0.000 |
| personal accomplishment | 3.53 ± 0.45 | 3.03 ± 0.4 |  | 0.952 |
| **Quality of life (points)** | | |  |  |
|  | 81.4 | 75.5** | **3869** | 0.000 |

Table 1. Comparison of depression. sleep disturbance. burnout and quality of life in the study subgroups

(** p<0.001)

| N=2540 | B | SE B | p | OR | CI 95%  Lower | CI 95%  Upper |
| --- | --- | --- | --- | --- | --- | --- |
| *Age** | 0.01 | 0.37 | 0.027 | 1.49 | 1.22 | 2.08 |
| Family status | 0.05 | 0.15 | 0.099 | 0.21 | 0.78 | 1.42 |
| Number of persons sharing a household | 0.02 | 0.15 | 0.086 | 0.22 | 0.76 | 1.37 |
| *Smoking** | 0.03 | 0.39 | 0.014 | 1.47 | 1.44 | 2.25 |
| *Drug consumption** | 0.95 | 0.48 | 0.003 | 1.91 | 1.15 | 1.99 |
| *Musculoskeletal disorders** | 1.12 | 1.57 | 0.041 | 1.38 | 1.14 | 3.71 |
| *Time spent ≥6 hours online** | 0.16 | 0.09 | 0.000 | 1.73 | 1.717 | 2.04 |
| *Daily time interval** | 1.32 | 1.94 | 0.006 | 1.72 | 1.56 | 1.96 |
| *Sleep disturbance** | 0.08 | 0.05 | 0.000 | 1.84 | 1.83 | 2.03 |
| *Burnout** | 0.14 | .042 | 0.009 | 1.8 | 1.16 | 1.94 |
| *Depression** | 0.01 | .013 | 0.001 | 1.97 | 1.77 | 2.02 |

Table 2. Factors independently associated with IA in a logistic regression analysis.
